# Framing Scientific Analyses for Risk Management of Environmental Hazards by Communities: Case Studies with Seafood Safety Issues

**DOI:** 10.1289/ehp.7655

**Published:** 2005-06-27

**Authors:** Nancy L. Judd, Christina H. Drew, Chetana Acharya, Todd A. Mitchell, Jamie L. Donatuto, Gary W. Burns, Thomas M. Burbacher, Elaine M. Faustman, Marine Resources for Future Generations

**Affiliations:** 1 Department of Environmental and Occupational Health Services,; 2 Institute for Risk Analysis and Risk Communication, and; 3 Center for Ecogenetics and Environmental Health, University of Washington, Seattle, Washington, USA; 4 Korean Women’s Association (Tacoma, Washington, USA), Indochinese Cultural and Service Center (Tacoma, Washington, USA), Tacoma–Pierce County Health Department (Tacoma, Washington, USA), Citizens for a Healthy Bay (Tacoma, Washington, USA), Washington Department of Fish and Wildlife (Olympia, Washington, USA), and Washington Department of Health (Tumwater, Washington, USA); 5 Swinomish Indian Tribal Community, Water Resources Program, La Conner, Washington, USA; 6 Department of Resource Management, University of British Columbia, Vancouver, British Columbia, Canada; 7 Shoalwater Bay Indian Tribe Environmental Laboratory, Tokeland, Washington, USA

**Keywords:** Asian and Pacific Islanders, case studies, communities, community-based participatory research, framing, risk management, seafood, tribal nations

## Abstract

Risk management provides a context for addressing environmental health hazards. Critical to this approach is the identification of key opportunities for participation. We applied a framework based on the National Research Council’s (NRC) analytic–deliberative risk management dialogue model that illustrates two main iterative processes: informing and framing. The informing process involves conveying information from analyses of risk issues, often scientific, to all parties so they can participate in deliberation. In the framing process, ideas and concerns from stakeholder deliberations help determine what and how scientific analyses will be carried out. There are few activities through which affected parties can convey their ideas from deliberative processes for framing scientific analyses. The absence of participation results in one-way communication. The analytic–deliberative dialogue, as envisioned by the NRC and promoted by the National Institute of Environmental Health Sciences (NIEHS), underscores the importance of two-way communication. In this article we present case studies of three groups—an Asian and Pacific Islander community coalition and two Native American Tribes—active in framing scientific analyses of health risks related to contaminated seafood. Contacts with these organizations were established or enhanced through a regional NIEHS town meeting. The reasons for concern, participation, approaches, and funding sources were different for each group. Benefits from their activities include increased community involvement and ownership, better focusing of analytical processes, and improved accuracy and appropriateness of risk management. These examples present a spectrum of options for increasing community involvement in framing analyses and highlight the need for increased support of such activities.

Risk management provides a context for addressing environmental health hazards. Critical to this approach is the identification of key opportunities for participation. Ideally, affected parties are involved early and throughout the decision process through continuing dialogue. The reality, however, often falls short of this ideal. The involvement of affected parties is commonly limited to community members’ being informed of the results collected and assessed by scientific experts and decision makers. Increasingly, communities are provided the opportunity to comment on documents or studies that are presented to them in near-final form, but rarely is community input used to frame and provide context at the outset of the studies themselves. Here we explore some of the opportunities and challenges of broader community participation within the theoretical structure of the risk-management paradigm. We begin by presenting a model of the analytic–deliberative risk-management framework, with an emphasis on framing activities in this structure, and then present three examples that illustrate community approaches to framing exercises. Using examples of activities by communities with concerns about seafood safety, we explore a range of options for increasing community involvement in shaping the scientific approaches used in risk management.

Our examples come primarily from established connections between University of Washington researchers and community partners. These connections originated or were developed more fully through the National Institute of Environmental Health Sciences (NIEHS) Center for Ecogenetics and Environmental Health’s town meeting, “Voices for Healthy Environments, Healthy Communities,” held in Seattle, Washington in September 2000. The NIEHS Center for Ecogenetics and Environmental Health (CEEH) researchers and staff interacted with > 300 participants, representing > 40 community groups, tribal nations, legislators, and agencies, in challenging discussions of race, poverty, and pollution. This was one of 16 town meetings supported by NIEHS across the country as part of NIEHS’s commitment to developing a research agenda responsive to community needs (O’Fallon et al. 2003). The case studies presented here provide lessons for expanding community participation in designing environmental health risk research questions (framing) under various circumstances including the rationale for community action, differences in resources, and involvement of scientific experts.

## Community Involvement in Analytic–Deliberative Risk-Management Dialogue

Involving affected parties at all major phases of the risk decision process is an important component of nearly all risk-management paradigms (National Research Council 1996, 2000a). Affected parties should be allowed to express their own needs and help shape objectives for risk management. However, involvement is challenging. One barrier to effective participation is not involving affected parties early enough in the process. This is often seen when fish contamination problems are addressed and when fish advisories are issued (Jardine 2003). Other barriers include information failing to reach communities, the lack of awareness of some environmental health issues, and the varying degrees of scientific understanding. Experience, skills, scientific training, and local knowledge and values can vary considerably among participants working on a decision process, and agencies may not have access to important local knowledge, may not understand what affected parties care about, or may not be aware of behaviors that affect exposure to contamination. Researchers need help with all these issues to appropriately address risk concerns.

The National Academy of Sciences (NAS) has repeatedly called for early, active, continuous, and transparent community involvement in risk-influenced activities (National Research Council 1996, 2000a, 2000b). *Understanding Risk: Informing Decisions in a Democratic Society* (National Research Council 1996) offers a detailed framework for improving complex decision processes. It describes an analytic–deliberative process, in which theories, results, and scientific analyses inform the deliberative processes used to discuss and determine the appropriate course of action. At the same time, the deliberative processes frame the scientific analyses. During the many decision phases, the participants (public officials, scientists, and interested/affected parties) interact and participate in the analysis and deliberation.

**Figure 1:**
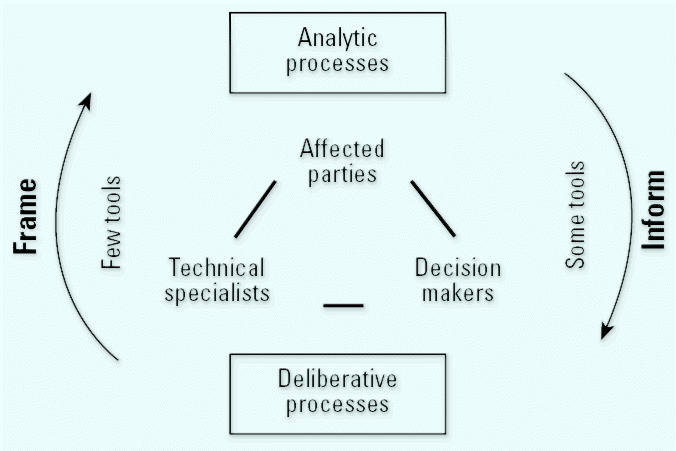
Model of the analytic–deliberative risk process adapted from Drew et al. (2003) and the National Research Council (1996).

To facilitate our implementation of the NAS framework, we adapted the original NAS framework to specifically highlight the interplay among the analytic–deliberative processes (Figure 1; Drew et al. 2003; National Research Council 1996). The trio of participants (affected parties, technical specialists, and decision makers) is fundamental to the process, and each group should participate in all phases. Moreover, individuals may participate as members of more than one group, depending on training, experience, and their role in the decision process. Little attention has been paid to the information needs inherent to the analytic–deliberative process (Drew et al. 2004). Generally, more attention has been given to the informing aspects than to the framing aspects, and more tools have been developed to support the analytic aspects of the processes than the deliberative aspects. As a consequence, participation in the framing process, especially by affected parties, is often limited.

Sometimes involvement activities are too focused on one-way information flow: from those who are making decisions (such as government agencies) to those who are being informed. Most involvement paradigms call for two-way information flow, but they offer few specific recommendations for facilitating this, particularly for increasing participation in designing research questions (Drew et al., in press). Various public participation models and tools offer opportunities to inform, consult, involve, collaborate with, and empower affected/interested parties [International Association for Public Participation (IAP2) 2000; Renn et al. 1995].

Community-based participatory research (CBPR) provides a tool for expanding community involvement in research projects and potentially for increasing participation by affected community members (O’Fallon and Dearry 2002). The NIEHS defines CBPR as a methodology that promotes active community involvement in the processes that shape research and intervention strategies and that promotes involvement in the conduct of research studies. The CBPR approach is designed to apply more generally to environmental health issues of concern, to ensure meaningful involvement by community members.

These CBPR principles of early and active community engagement also apply to increasing community involvement in all aspects of the analytic–deliberative risk dialogue. An advantage of considering environmental health issues in a risk context is that risk-management science is directed toward providing information for decision making and dealing with uncertainties (Faustman and Omenn 2001; Morgan and Henrion 1990). The principles of CBPR can be achieved more easily when the analytic–deliberative approach is applied in its ideal form (i.e., when all interested and affected parties are involved in informing and framing processes). Using example case studies, we discuss options for moving beyond processes that simply inform affected communities to processes that involve communities in framing relevant scientific questions.

## Informing

Informing makes information from analytic processes (often scientific or research) accessible to all parties, so community members may more fully participate in deliberative (risk-management) discussions. In the context of fish contamination issues, community involvement is often limited to informing activities. There is a growing literature describing, evaluating, and improving these activities, most related to the issuance of fish advisories (Burger et al. 2003; Connelly and Knuth 1998; Jardine 2003; Knuth et al. 2003; Shubat et al. 1996). A common theme from many of these studies is the need for two-way communication and earlier involvement by communities. The analytic–deliberative dialogue (Figure 1) is an iterative process and can be flexible as new information becomes available and new participants join the process.

Issuing fish advisories is often not an iterative process, however. Once advisories are issued, the public is wary of reusing a fishing resource that once was declared unsuitable (Jardine 2003). The issuance of advisories tends to be a top-down process, as decisions about acceptable risks and alternatives are often made without including affected parties. Such top-down processes may not be appropriate for all consumption and cultural groups [Columbia River Inter-Tribal Fish Commission (CRITFC) 1994; Sechena et al. 1999; Shubat et al. 1996; Suquamish Tribe 2000; Toy et al. 1996]. This is reflected in the increasing number of fish advisory evaluations calling for early involvement (Jardine 2003). In more advanced models, information flow is two-way but is still limited to informing activities such as risk communication about fish contamination (Burger et al. 2003; Jardine 2003; Knuth et al. 2003). This might include community partners developing fish advisories (informing) without being involved in the scientific analysis used to shape the advisory (framing) (Figure 1). Without real meaningful involvement during the framing steps, informing processes will not be as significant to affected communities.

## Framing

Framing allows concerns that arise through deliberative processes to shape analyses (Figure 1). This presents the potential for major expansion of community involvement in the risk-management process. In particular, there are many such possibilities in risk management of contaminated fishing resources.

As noted previously, efforts in framing activities have been limited (Jardine 2003; Knuth et al. 2003). Reasons for this can include a lack of communication among community groups, technical specialists, and decision makers, leading to nontransparent decision processes (Drew et al. 2004, in press). In other words, how do researchers and decision makers select a research agenda or a decision process after environmental hazards or issues are recognized? Another reason affected parties are not involved in framing research more often is that there are limited funds dedicated to support involvement up front.

There are several benefits of expanding participation in framing research questions for all parties involved in the analytic–deliberative dialogue. Community participation may result in the design of more effective analyses (Bierle 2002; Drew et al., in press; Israel et al. 2001). This participation may also promote research addressing community needs, community acceptance of the processes, understanding of environmental health risks, and informed behavior changes (Jardine 2003). Moreover, community involvement in framing may increase overall dialogue and thereby improve informing processes essential to risk management.

In our experience, more effort has been focused on informing than on framing risk questions and risk management activities (Drew et al. 2004, in press; Judd et al. 2003b; Polifka and Faustman 2002). Our objective here is to report several community framing activities that have shaped how analytical processes (research) will be carried out to assess the safety of fish consumption. By exploring similarities and differences across the three examples, we hope to present a range of framing approaches that may also be appropriate for other groups.

## Case Studies of Communities Involved in Framing

We have had the privilege of collaborating with several dynamic communities that are proactively addressing their environmental health concerns. Here we highlight their efforts in framing aspects of the analytic–deliberative risk-management process. Common themes across these examples, including challenges and benefits, are explored using a case-study approach (Yin 1994). These descriptive case studies document collaboration between university researchers and community, tribal, and agency partners. The three case studies describe interactions with Marine Resources for Future Generations, the Swinomish Indian Tribal Community, and the Shoalwater Bay Indian Tribe. These interactions have been through participation on advisory boards, and the importance of relationship building has been key. All three groups are located in Washington State, and the importance of fish and seafood in each is high. Recent seafood consumption surveys indicate that average tribal and Asian and Pacific Islander (API) community members consume 3–10 times the amount of fish and shellfish of average U.S. consumers [Sechena et al. 1999; Suquamish Tribe 2000; Toy et al. 1996; U.S. Environmental Protection Agency (EPA) 1997a]. High-end tribal consumers may eat 20 times the amount of average U.S. consumers (Suquamish Tribe 2000; U.S. EPA 1997a). In addition, sources and types of fish and shellfish consumed differ from community to community (Judd et al. 2004). Traditional diets and reliance on subsistence fishing/harvesting contribute to the higher consumption rates of tribal and API community members.

Each of these groups has concerns about specific contaminants (e.g., polychlorinated biphenyls, biotoxins, pesticides, and methyl-mercury) in seafood they eat regularly. Our previous studies indicated that the specific collection, preparation, and consumption practices of tribes and API communities may place them at greater exposure to some contaminants. Additionally, our studies have shown that monitoring practices by some regulatory agencies may not be sufficient to evaluate or protect these vulnerable groups from the potential health risks (Judd et al. 2003a,b).

Each community has its own story of how their efforts to address potential health risks from consuming contaminated seafood began and how they eventually became active in framing activities.

### Marine Resources for Future Generations.

The Marine Resources for Future Generations (MRFFG) program began in 1997 in Pierce County, Washington. The initial mission of the group was to ensure the safe and wise use of seafood resources and compliance with state regulations, such as licensing and appropriate shellfish collection, by API communities in the county. The group includes two social service organizations: the Korean Women’s Association (KWA), which serves the Korean, Samoan, and Filipino communities, and the Indochinese Cultural and Service Center (ICSC), which serves the Vietnamese, Cambodian, and Laotian communities. Government agencies and nongovernmental partners provide support and educational resources and make MRFFG a strong coalition. The connection with the University of Washington (UW) was made at the NIEHS town meeting during a seafood safety break-out session, and UW staff has since attended the monthly meetings, provided technical advice, and assisted in MRFFG projects.

For many API communities, seafood is an important part of both nutrition and cultural traditions, making seafood safety a very pressing matter. The Washington Department of Fish and Wildlife (WDFW) was concerned that their usual methods of education (multilingual brochures and signs) were not reaching many API community members. The MRFFG group began when KWA and ICSC joined with WDFW to address illegal harvesting issues, including shellfish collection from closed and contaminated beaches. Other partners soon joined, and over the years the group’s efforts have expanded to include many other issues including non–point-source pollution, mercury in fish, and invasive species. An initial condition for participating agencies and organizations is a long-term commitment, not just a pilot project effort. This has been key to the success of the group that has held monthly meetings for the last 7 years, even as grant support has waxed and waned.

Early on, MRFFG’s educational outreach found that the sources of seafood sold at local markets were unknown. This was an important issue for the group to address. As community members began to understand that some beaches were not safe for harvesting shellfish, they wanted to know the source of the seafood they purchased in markets. At the same time, the group was concerned that education about local contamination had led people to believe that seafood from anywhere else (besides local contaminated beaches) would be cleaner. MRFFG launched its own effort to investigate the sources of local seafood. This project is an excellent example of community-driven framing of problems in the risk-management process because these efforts focused on developing and pursuing scientific questions to better understand potential health risks.

The main goal of the project was to talk with local vendors and determine the sources of their seafood. If the seafood was local, they wanted to know specifically which beach it was from and whether it was legally harvested, as well as the sources of imported seafood. Another goal of the project was to provide education about the health importance of regulations for collection and sale of seafood to vendors. The businesses were all within API communities, and MRFFG wanted to support these businesses by providing them with information to help ensure community health, which would ultimately also benefit retailers. MRFFG’s multilingual youth administered the surveys in a nonthreatening manner, collecting information, not enforcing regulations.

Fourteen youth participated and visited 10 stores in Tacoma and Seattle, serving mostly Korean, Vietnamese, Cambodian, Samoan, and Filipino community members. Results indicated that the stores were importing from overseas most of the fish they sold, and this choice was driven by both customer and owner preferences. Seventy percent were aware of health dangers related to seafood, but at least 20% of the stores had no awareness of health dangers associated with shellfish contamination or illegal harvesting. MRFFG concluded that they needed to increase awareness of seafood safety issues to ensure community health. This process began with providing literature from the WDFW and the Washington Department of Health. Thus, this framing and analysis project fed into an informing process in an iterative way and expanded community involvement.

MRFFG continues educational efforts with local shopkeepers to ensure the safety of the seafood they sell. They have also begun investigation and education efforts with stores about the environmental dangers of importing invasive species. These community-driven efforts have promoted community health while encouraging community businesses. Outside groups, even those fluent in Asian languages, could not have performed this investigation and education process as effectively as the youth because the business owners might have perceived a threat (in the form of an enforcement action), and they might not have provided information.

Funding for MRFFG projects, such as this one, have come from a variety of sources, including U.S. EPA headquarters and Region 10, regional foundations, the Puget Sound Water Quality Action Team, the Russell Family Foundation, and several MRFFG member organizations. The group has also successfully obtained funds through competitive processes geared primarily toward community organizations. Despite funding being an annual uncertainty, MRFFG has effectively leveraged their resources to address community seafood safety concerns. The group’s longevity rests in the continued commitment of its members that extends beyond the funding period of one grant or project. The efforts of MRFFG also demonstrate that community groups with limited resources can engage in framing activities that empower them to make more educated decisions about managing environmental health risks.

### Shoalwater Bay Indian Tribe.

The Shoalwater Bay Indian Tribe is concerned about the potential impacts of environmental quality on their health for several reasons. The Shoalwater Bay Indian Reservation (SBIR) is located on Willapa Bay, in the most isolated rural area of northern Pacific County in Washington State. The tribal community includes just 237 people (Shukovsky 2002). Fish and seafood are major dietary components for the Shoalwater; these resources have very important traditional and spiritual roles in tribal communities (CRITFC 1994; Suquamish Tribe 2000; Toy et al. 1996). Although a small tribe, the Shoalwater must deal with a large variety of environmental issues. One of the biggest of these is the widespread commercial use of pesticides on lands surrounding the reservation. Diazinon has been sprayed over the nearby cranberry bogs to kill fire worms, which destroy the plants. Railroad ties, heavily treated with a fungicide to prevent rotting, are situated throughout the bogs. The pesticide carbaryl is applied to the many oyster beds around Shoalwater Bay (and Willapa Bay, a larger connected body of water) in an effort to retard ghost shrimp populations. The tideflats are also sprayed routinely with glyphosate to control *Spartina*, a destructive weed. Other environmental concerns include the presence of fecal coliform and marine biotoxins. Harmful algal blooms that release biotoxins, such as saxitoxin and domoic acid, have led to several recent beach closures for shellfish harvesting [Commission on Asian Pacific American Affairs (CAPAA) 2004; Washington Department of Health (WADOH) 2004]. Additionally, many septic systems on or adjacent to the reservation are failing (Laundry Alternative 2004; Puget Sound Action Team 2004). All of these factors may affect shellfish quality.

In the mid-1990s, the U.S. EPA conducted several environmental assessments (water, air and soil quality) in the region (U.S. EPA 1997b). These investigations, made in response to a high prenatal and neonatal mortality rate within the Shoalwater Tribal community, have been limited in scope. The final report recommended further testing at additional sample sites to provide more complete information (U.S. EPA 1997b).

In September 2000, Shoalwater leaders attended the CEEH’s town meeting and voiced their concerns to NIEHS Director Kenneth Olden. As a result of this meeting, the NIEHS provided support to enable the CEEH’s Community Outreach and Education Program (COEP) and the Shoalwater’s Environmental Division to work together. This effort represents one of many projects implemented by Shoalwater’s Environmental Division, most of which are administered and managed internally. Their new on-site environmental laboratory has increased the ability of the tribe to engage in many framing and analytical activities independently to address their environmental health risk concerns. Additionally, to holistically address health concerns on the reservation, the Shoalwater constructed a new health clinic and have developed intensive prenatal care and well-baby programs.

The Shoalwater, in collaboration with COEP and the Institute for Risk Analysis and Risk Communication (IRARC), has used NIEHS support to engage in framing tribal environmental concerns. The Shoalwater developed a seafood consumption survey tool and a shellfish quality management plan. Both the shellfish plan and the survey tool were included in a proposal submitted to the Administration for Native Americans (ANA) that has since received funding. The ANA project described monitoring subsistence food species that are consumed by tribal members for environmental contaminants. This approach was favored by most tribal members, who were surveyed using a pilot seafood-consumption survey tool. The results will be used to create a prioritized list of the species to be tested for contaminants. The results of these tests will be incorporated into the tribal management plan to assess the shellfish quality in Willapa Bay. The Shoalwater Tribe is also awaiting response on other research proposals submitted to U.S. EPA and NIEHS. These include studies to look at seafood contamination in the context of other dietary risk factors and, when funded, will use technical contacts at the University of Washington.

The Shoalwater have faced many difficulties, but they have maximized their resources to address their concerns. Proposal development can be a daunting task, particularly for communities with many competing priorities and limited technical, material, and human resources. The Shoalwater Tribe has successfully developed competitive proposals that will enable them to more fully frame and analyze their environmental health risk concerns.

### Swinomish Indian Tribal Community.

The Swinomish Tribe’s research project, Bioaccumulative Toxics in Native American Shellfish (BTNAS), is another example of a tribal community framing their own questions. The Swinomish Reservation is located on the shores of central Puget Sound and is home to 1,000 Native Americans, of whom 700 are enrolled Swinomish members. Swinomish Tribe members are concerned about environmental contamination threatening their traditional use of resources, particularly shellfish. There are numerous potential sources of contamination within a mile radius of the reservation, including petrochemical and industrial facilities, landfills, municipal sewer outfalls, two marinas, two boatyards, log storage facilities, and agricultural land treated with pesticides and fertilizers. The Swinomish Tribe has initiated investigation into the potential contamination of water, sediments, and shellfish. The purpose of the project is to ensure safety and promote continuation of healthy, traditional lifestyles and/or to begin proactively addressing cleanup and mitigation of contaminated sources. The Swinomish Tribe requested that a screening study of contaminants be performed in Padilla and Fidalgo Bays by the Washington State Department of Ecology. The initial study indicated the presence of numerous persistent pollutants, including arsenic and polychlorinated dibenzofurans (PCDFs) (Johnson et al. 1997). Later studies indicated the need for additional sampling to understand the magnitude and the health implications of the contamination (Johnson 1999, 2000).

Shellfish contamination represents one of a number of threats to the Swinomish maintaining their traditional lifestyle. It is extremely important to the Swinomish that the effort to investigate the contamination and potential health risks be performed by the Swinomish Tribe. The Swinomish have significant internal resources, including several environmental scientists with advanced degrees, an on-site chemistry lab, and an ongoing shellfish monitoring program funded by the U.S. EPA and the Bureau of Indian Affairs (BIA), primarily for paralytic shellfish monitoring. Moreover, this is an issue of sovereignty. The Swinomish Tribe prefers to control how such a study is conducted to ensure that it addresses (frames) the Swinomish Tribe’s environmental health concerns and that the information gathered is used and interpreted by the Swinomish Tribe.

In summer of 2000, the Swinomish Planning Office and their intern (funded by the Environmental Careers Organization) developed the BTNAS proposal. Although the Swinomish Tribe possessed the infrastructure required to develop an in-depth environmental sampling, analysis, risk management, and education plan with a significant cultural component, they were unfamiliar with the complexities of a federal grant application. The Swinomish sought help with this technical challenge at CEEH. Additionally, at the NIEHS town meeting, the Swinomish Tribe submitted their concerns related to the difficulties of the grant proposal procedure for communities unfamiliar with the federal funding process. Providing feedback to agencies that clearly have a mandate and desire for community-based research should make it easier for communities with the capacity to receive grants directly.

With final approval from the Swinomish Tribe’s governing council, the grant was submitted and received favorably by the NIEHS, but was not funded. It was, however, recommended to the U.S. EPA, and in 2002, the Swinomish Tribe was awarded the largest-ever U.S. EPA research grant to a tribal nation. The Swinomish Tribal Planning Office had the core staff and resources to take on a project of this magnitude, in addition to many other ongoing water quality projects. The project necessitated hiring new staff for the many new responsibilities and activities. Currently, IRARC and COEP researchers act as advisors to the BTNAS project and have assisted and/or acted as principal investigators for subsequent grant applications. So far the BTNAS project has collected two seasons of field samples, and sample analysis is in progress. The planning office staff has been annually updating the Swinomish General Council on the progress of the BTNAS project. The Swinomish Annual Report and the free monthly tribal newsletter, *Keeyoks*, provide information to tribal members about BTNAS project developments. Additionally, the Swinomish environmental education program works in the public schools, providing outreach and education on local environmental health issues.

More recently, the Swinomish organized a meeting of environmental scientists from several nearby tribes to discuss common concerns, upcoming funding opportunities, and approaches for sharing resources. This meeting was significant in that it was organized by the tribes, for the tribes. The BTNAS project has also been presented at several scientific meetings.

The BTNAS project is another good example of a community framing their own environmental health questions. To pursue the specific questions of the Swinomish Tribe about the condition of the local environment and safe consumption of shellfish, a technical approach is needed. The Swinomish Tribe has the resources to develop a plan, obtain funding, and pursue these questions. Because the Swinomish Tribe developed the plan, it addresses their needs while maintaining tribal sovereignty through tribal control of research activities, findings, and interpretation. The Swinomish Tribe Planning Office is in an optimal position to inform the tribal community about the project and incorporate community feedback for framing future activities. The ongoing activities illustrate how the Swinomish Tribe is using information from this research to evaluate their risks from shellfish exposure.

## Summary of Community Experiences with Framing Activities

A challenge for researchers is determining how to work with communities to understand how their questions are framed and how to incorporate this process in their research programs. This challenge has been identified in previous work, such as involving communities in risk-management processes related to cleanup and transportation of nuclear waste (Drew et al. 2003, in press). In that case and the case examples presented here, the challenges are unique to each situation and require significant time investments and resources for the communities and the collaborators. This has also been identified through numerous CBPR projects (O’Fallon and Dearry 2001, 2002; O’Fallon et al. 2003; Seifer 2000; Thompson et al. 2001). The examples of efforts by MRFFG, the Swinomish Tribe, and the Shoalwater Bay Indian Tribe illustrate a range of opportunities for communities in framing activities. Each community had different issues and approaches, including who was involved, how the effort was financed, and the types of outcomes. The various outcomes are summarized in Table 1, and the many common themes that the groups shared are described in Appendix 1.

The MRFFG project presents a grass-roots approach to addressing community problems. After embarking on an educational effort (informing) to reduce community exposures to contaminants in locally collected shellfish, the group recognized the importance of assuring the safety of seafood at local markets. This work grew out of their original mission, which had not included investigatory work. However, as the group framed the question of local markets’ sources of seafood, they found that they lacked information. Undaunted, they took the initiative and pursued the information themselves (Table 1). This was done primarily by leveraging limited funds from government and private sources. MRFFG drew on expertise and support from all its members: community youth, elders, county and federal agencies, and nonprofit and academic partners. This example demonstrates that groups that do not typically perform scientific investigations can perform framing activities and that framing and analysis can be done with limited resources if the group has a strong commitment to addressing the question. By internally carrying out the study, the community has ownership of the activity and can better facilitate community education and dialogue about the results. Developing and pursuing these questions internally fosters community interest, support, and positive action to address problems.

The Shoalwater Bay Indian Tribe’s effort to develop a proposal to investigate contaminated shellfish represents a very different approach that began with support from government agencies (NIEHS) and collaboration between their own scientists and outside scientific experts. The Tribe engages in many research efforts to ensure a healthy community. In this particular example, the Tribe investigated potential shellfish contamination in collaboration with outside researchers (Table 1). This preliminary investigation, itself a framing exercise, was used in several subsequent research proposals, some that have been funded and some that are pending. Thus, the community was able to obtain support, both financial and technical, specific for its framing efforts. This has led to the development of successful research proposals specific to the Tribe’s questions and concerns.

The Swinomish Tribe has had an ongoing shellfish-monitoring program, but this was not adequate to address concerns about bioaccumulative toxicants in shellfish. The Swinomish Tribe obtained funding and is currently engaged in research including iterative framing of questions about shellfish contamination (Table 1). It has been paramount for the Swinomish to have tribal autonomy over the scientific questions asked, project execution, data collection, and data interpretation. The information collected will be used to evaluate current and future risks from shellfish exposures. The Tribe received some help from academic researchers with the grant application process, in addition to technical and outreach expertise.

Thus, the examples presented here demonstrate a range of possibilities in terms of the questions asked, the way they were formulated and pursued, how experts were involved, and how they were funded. Some projects leveraged limited funds from a variety of sources to pursue their concerns, and some obtained resources specifically for framing questions, which they then used for research and/or in developing more complete proposals. Tribes are in a unique situation with regard to applying for funding in that they, as sovereign nations, often have more developed infrastructures than many community groups. They are also eligible for some tribal-funding sources (e.g., BIA and U.S. EPA) that cannot be pursued by other communities.

Despite many differences in their problems and approaches, many common themes emerged from the experiences of these communities (Appendix 1). Some common benefits of framing that are shared across the groups include research that better meets community needs and increased community ownership. These examples also show how framing can help build internal knowledge and capacity. For all the groups, environmental issues are among many competing issues, and the process of framing may be outside the usual scope of the group’s activities. Finally, trust and connections beyond the community may also be needed, and the process of framing may develop as many or more questions than it addresses. These commonalities highlight benefits and challenges that may be part of framing by other communities and can be helpful in determining the potential utility of the process and in anticipating some of its difficulties.

## Conclusion

This article has presented three case studies of successful community action in framing scientific analyses of environmental health risks. We used the NRC’s analytic–deliberative process to think about the different components needed for CBPR in a risk context. The analytic–deliberative process prompts us to pay special attention to roles communities can play in both framing research questions and in informing and educating all parties involved in the risk process. Framing is an integral part of the analytic–deliberative risk process and can open important opportunities for two-way dialogue and communication among researchers and community/tribal partners. Few accounts in the literature have shown how this happens and why. We have presented three case studies related to seafood safety that illustrate how the framing process can work. The efforts of these case study groups and their partners have opened opportunities and empowered them to address their environmental health risk concerns.

The case studies present a range of possibilities for communities to be involved in framing activities. These projects span different environmental problems with communities using a variety of approaches, including how (or if) outside experts were involved and how the effort was funded. There were elements of framing and informing in each of the examples, demonstrating the interconnectedness and importance of both. Many common themes from their experiences emerged, including how framing helped in capacity building, how they balanced competing concerns, and how the communities benefited. However, given the pressure to deliver maximum production for grant dollars spent, there is little incentive for researchers and agency staff to engage in activities that are not mandated, may not be recognized as results, and are likely to be time and resource intensive. Increasing community and tribal participation through framing and CBPR requires significant investments of time and resources by all the collaborators. Given the value of this broader involvement, funding agencies should recognize, encourage, and even mandate community involvement to specifically frame and address environmental health risk issues. This research direction will ultimately lead to more relevant and realistic environmental health risk management solutions.

## Figures and Tables

**Table 1:** Summary of case study framing activities and outcomes

Group	Issue	Framing activity	Outcome
MRFFG	Concern about the sources of seafood and seafood safety at community stores	Community youth interviewed local merchants	Better characterization of seafood sources and improved understanding of potential exposure and risks from these
Shoalwater Bay Indian Tribe	Concern about local shellfish contamination’s impact on community health	Developed an assessment plan and submitted a grant proposal to fund research	Obtained funding to sample shellfish for contaminants and to perform seafood consumption surveys
Swinomish Indian Tribal Community	Concern about local shellfish contamination’s potential effect on current and future resource use	Expanded existing infrastructure for shellfish monitoring to include bioaccumulative biotoxicants	Data collected is being used to evaluate current and future risk from shellfish exposures

## Appendix 1. Common Themes from Framing Exercises

Benefits

- Community framing better addresses local environental health risk concerns
- Community framing leads to better communication of research processes and findings
- Greater incentive for community framing if community and tribes are involved in data analysis
- While outside resources are useful, it is important that the community maintains ownership and that framing is driven by local needs Capacity building
- Application processes to obtain funding are challenging for groups not traditionally involved in grant writing
- Internal community/tribal technical resources may be limiting; framing processes may help build organizational capacity
- Long-term commitment by all partners is required for framing processes to succeed Competing issues
- Environmental health risks are among many competing community priorities
- Framing processes (e.g., developing environmental risk questions and/or proposals and actions to pursue them) may be outside the scope of general activities of many organizations Other
- Importance of trust with collaborating partners (regarding commitments to partnerships, tribal sovereignty, or enforcement issues)
- Interconnections within small communities facilitates communication and participation
- Framing is an expanding process and often leads to more questions
